# Spectrum of haemoglobinopathies diagnosed by cation exchange-HPLC & modulating effects of nutritional deficiency anaemias from north India

**DOI:** 10.4103/0971-5916.73390

**Published:** 2010-11

**Authors:** Seema Rao, Rakhee Kar, Sanjeev Kumar Gupta, Anita Chopra, Renu Saxena

**Affiliations:** *Department of Hematology, All India Institute of Medical Sciences, New Delhi, India*

**Keywords:** Diagnosis, haemoglobinopathies, HPLC, nutritional deficiency anaemia, thalassaemia

## Abstract

**Background & objectives:**

The usefulness of cation exchange high performance liquid chromatography (CE-HPLC) as a tool for detection of thalassaemia/haemoglobin variants was evaluated in a prospective study in a tertiary care centre in north India. We also tried to evaluate the effect of concurrent nutritional deficiency on the HPLC pattern in the local ethnic population.

**Methods:**

A total of 800 blood samples were analyzed on the Bio-Rad Variant HPLC system by β-thal short program. The retention times, proportion of the haemoglobin (%), and the peak characteristics for all haemoglobin fractions were recorded. Alkaline and acid haemoglobin electrophoresis was performed to document the identities of the haemoglobin variants, wherever necessary. Many cases were subjected to family studies for a definitive diagnosis.

**Results:**

Among 800 samples tested, 553 (69.1%) were found to have normal HPLC pattern. Apart from β- thalassaemia, nine additional variants were encountered; HbS (2.8%), HbE (2.5%) and HbD (1.1%) being the most common variants present. Other variants included Hb Q-India, Hb-Lepore, δβ-thalassemia/ HPFH, HbD-Iran, HbJ-Meerut and HbH disease. There was a significant decrease in the level of HbA_2_ associated with iron deficiency anaemia (IDA) (*P*=0.004) and increase in megaloblastic anaemia (*P*<0.001) among subjects with normal HPLC pattern.

**Interpretation & conclusions:**

HPLC was found to be a simple, rapid and reliable method for the detection of hemoglobin variants. An accurate diagnosis can be provided in majority of cases by use of retention time, proportion of total haemoglobin, and peak characteristics of HPLC. Haemoglobin electrophoresis and family studies play a valuable role in difficult cases. Concurrent nutritional deficiency also has an effect on HbA_2_ levels.

The laboratory diagnosis of thalassaemias and other haemoglobinopathies can be achieved by a step-wise approach starting with a detailed clinical history, thorough haematologic evaluation [including haemoglobin level, complete blood count (CBC), reticulocyte count, and red blood cell (RBC) morphology], protein based analytic methods [alkaline and acid Hb-electrophoresis, isoelectric focusing (IEF) and high performance liquid chromatography (HPLC)] and nucleic acid based methods [such as polymerase chain reaction (PCR), reverse transcriptase (RT)-PCR, and sequencing of genomic DNA]^1^,^2^. Family studies also play a crucial role in clinching the diagnosis in certain problematic cases.

The aim of the present study was to evaluate the role of cation exchange HPLC (CE-HPLC) along with adjunctive tests as needed in the diagnosis of thalassaemias/haemoglobinopathies and to see the profile of these in the indigenous population. It is especially important to validate the role of HPLC, as it is less labour intensive with rapid turn around time and better reproducibility compared to Hb electrophoresis. Moreover, it can replace tedious procedures like estimation of foetal haemoglobin and HbA_2_ quantitation by column chromatography. We also tried to evaluate the effect of nutritional deficiency anaemia, especially iron deficiency anaemia (IDA), on the level of HbA_2_ in non-thalassaemia subjects and if the change was sufficient enough to confound the diagnosis of β-thalassaemia trait.

## Material & Methods

This was a prospective study carried out in the department of Hematology, All India Institute of Medical Sciences (AIIMS), New Delhi, over 7 months duration from January to July 2008. Consecutive 800 blood samples sent for suspected thalassaemia/haemoglobinopathy work-up were analyzed by CE-HPLC (BioRad laboratories, California, USA) using Variant β-thal short program. This included mainly transfusion requiring children and adults, antenatal cases and their family members. Cases with nutritional deficiency anaemia, where a co-existent thalassaemia/haemoglobinopathy was suspected, were also screened. No absolute exclusion criteria were used but for patients requiring blood transfusions, sampling was deferred for at least 4 wk after or just before next transfusion. Written consent was taken from all patients for using their sample for research purpose. About 1-2 ml of blood sample was collected in EDTA vial and was analyzed in automated cell counter (Sysmex XT 1800i; Kobe, Japan) for complete blood counts. Samples were stored at 4-8°C and were analyzed in batches within one week. Wherever required, haemolysate preparation was run on agarose gel electrophoresis at alkaline pH of 8.6 from the same sample. The presence of HbH was confirmed by using brilliant cresyl blue test for HbH inclusions. IDA was diagnosed on the basis of serum iron levels, total iron binding capacity, transferrin saturation and serum ferritin levels. Patients suspected of megaloblastic anaemia on account of a raised mean corpuscular volume (MCV>110fl), were confirmed mostly by bone marrow examination or serum vitamin B12/folic acid assay in some of the cases. This was a single time study and no follow up was done.

Continuous variables were expressed as mean±SD. Categorical variables were expressed as frequencies and percentages. Two sample t-test was applied for significance of association between continuous variables. All statistical analysis was performed using STATA 9.1 statistical software (Statacorp, College Station, Texas, USA).

## Results

*Samples:* Of the 800 subjects tested, 259 (32.4%) were males and 541 (67.6%) were females. The age range of patients was from 5 months to 77 yr with a median age of 25 yr (mean age 24.2 yr). The preponderance of young female patients was due to antenatal patients coming for routine HPLC work-up. Of the total cases, 553 (69.1%) were found to have normal HPLC pattern. Analysis of the retention times and %Hb for HbA_0_, HbA_2_, and HbF showed no significant difference with regard to column and/or reagent changes. In this study the influence of haemoglobin stability testing and patient ethnicity were not included.

*Retention times and proportions of haemoglobin variants:* The number of observations, %Hb fraction, haemogram findings and the type of Hb variants encountered are shown in the Table. Separation of cases into transfused and not transfused was not done. Although the sampling was done with due precaution, the HbA and HbF levels shown in the Table may not be truly representative particularly in the thal major/intermedia group due to frequent transfusion requirement in some of them. As expected, out of 800 samples, β- thalassaemia trait was detected most frequently *i.e*., in 145 (18.1%) cases with cut-off value of HbA_2_ being >3.9 per cent^3^ and β- thalassaemia major/ β- thalassaemia intermedia was seen in 23 (2.9%) cases. Apart from β-thalassaemia, nine additional variants were encountered; HbS (2.8%), HbE (2.5%) and HbD-Punjab (1.1%) were the most common variants present. Other variants included HbQ-India, Hb-Lepore, δβ-thal/HPFH, HbD-Iran, HbJ-Meerut, Hb-H disease. Possibility of an α-thalassemia trait could be suggested in 11 (1.4 %) cases on the basis of red cell indices and the levels of HbA_2_ [2.2 (± 0.3)%] which were significantly (*P*<0.001) lower than normal population 2.9 ± 0.4. However, results of further molecular testing were not available for a definite conclusion.

**Table:** Relevant laboratory parameters in relation to normal and abnormal haemoglobin variants

Presumptive HPLC Diagnosis	No. of cases (%)	Hb (g/dl)	MCV (fl)	MCH (pg)	MCHC (%)	RBC count (x10^6^/μl)	RDW-SD (%)	Hb A (%)	Hb F (%)	HbA_2_ (%)	Variant Hb (%)
Normal	553 (69.1)	9.6 (2.7)	83.3 (10.7)	25.1 (4.9)	30.6 (3.8)	3.82 (0.8)	47.6 (10.4)	87.4 (4.2)	0.2 (0.5)	2.9 (0.4)	
β-Thal trait	145 (18.1)	10.3 (2.1)	68.6 (7.4)	20.5 (2.6)	28.3 (1.8)	5.06 (0.9)	35.7 (8.1)	83.2 (2.1)	1.0 (1.3)	5.5 (0.6)	
β-Thal major/Intermedia	23 (2.9)	5.4 (1.7)	74.9 (8.5)	23.3 (3.7)	31.1 (3.2)	2.41 (0.9)	55.3 (14.4)	39.2 (30.5)	52.5 (35.2)	3.7 (1.5)	
Sickle cell trait	11 (1.4)	11.6 (1.8)	84.3 (3.4)	26.7 (2.3)	31.7 (2.3)	4.45 (0.54)	45.3 (16.2)	52.7 (3.8)	2.3 (3.6)	3.3 (0.3)	36.3 (4.8)
HbS/β-thal	6 (0.8)	7.6 (1.2)	75.2 (9.0)	21.8 (2.0)	29.2 (2.6)	3.49 (0.6)	49.5 (15.5)	4.6 (1.8)	18.3 (8.4)	4.5 (0.6)	71.7 (5.8)
Homozygous Sickle cell ds	4 (0.5)	8.3 (1.9)	90.5 (9.7)	29.5 (3.4)	32.7 (0.9)	2.8 (0.6)	66.8 (6.6)	8.5 (13.7)	18.3 (8.2)	2.4 (0.5)	74.5 (6.7)
HbE-trait	9 (1.1)	11.4 (2.3)	82.3 (4.4)	26.2 (1.5)	31.6 (1.1)	4.3 (0.7)	41.1 (5.8)	61.9 (1.9)	1.6 (3.7)	--	27.8 (3.4)
HbE/β-thal	10 (1.3)	6.2 (1.1)	63.9 (7.7)	19.3 (2.7)	30.1 (1.4)	3.2 (0.54)	47.9 (9.8)	18.1 (22.4)	21.7 (12.2)	--	52.3 (17.6)
Homozygous HbE ds	1 (0.1)	7.5	63	20.4	32.3	3.68	31.4	3.7	4.5	--	78.2
HbD-Punjab trait	7 (0.9)	8.1 (2.4)	70.3 (16.1)	20.4 (7.4)	28.3 (3.9)	3.98 (0.42)	42.3 (12.5)	55.7 (4.1)	0.5 (0.4)	1.7 (0.4)	34.6 (4.1)
HbD/β-thal	1 (0.1)	13.3	67.1	21.1	31.5	6.29	34.1	3.7	2.3	4.7	83
Double hetero HbSD ds	1 (0.1)	8.8	90.2	27.8	30.9	3.16	60.8	2.2	8.6	3.2	41.9/41
HbQ-India trait	3 (0.4)	12.5 (1.6)	80.7 (7.2)	26.6 (2.1)	32.9 (0.4)	4.69 (0.2)	37.9 (1.1)	71.5 (1.7)	0	2.0 (1.7)	18.5 (0.2)
Hb-Lepore trait	1 (0.1)	9.9	72.2	22.9	31.7	4.32	32.3	70.5	4.4	--	16.4
α-thal trait	11 (1.4)	7.6 (1.9)	63.8 (6.9)	16.8 (2.8)	26.3 (2.6)	4.66 (0.9)	39.0 (11.7)	85.9 (3.4)	0.3 (0.4)	2.2 (0.3)	
δβ-thal trait	6 (0.8)	11.1 (2.2)	69.0 (7.7)	20.8 (2.7)	30.1 (0.9)	5.36 (1.0)	40.7 (7.2)	72.5 (2.4)	13.3 (4.3)	2.8	
HPFH	(0.1)	11.1	73.5	23.9	32.6	4.64	39.9	62.4	31.5	2.7	
HbD-Iran trait	3 (0.4)	7.4 (4.3)	64 (13.7)	17.7 (7.1)	26.9 (4.8)	3.99 (0.8)	32.6 (3.8)	49.6 (1.9)	0.8 (0.3)	--	40.6 (2.6)
Hb-H disease	1 (0.1)	4.9	84.0	20.7	24.6	2.3	45	87.7	0.4	2.2	--
HbJ-Meerut trait	1 (0.1)	11.9	91.1	30.1	33.1	3.95	45.9	65.4	0.5	2.5	26.5

All figures mentioned are mean ± SD. Two cases with high HbA1c are not mentioned in this Table. Hb, haemoglobin; MCV, mean cell volume; MCH, mean cell haemoglobin; MCHC, mean cell haemoglobin concentration; RBC, red blood cell; RDW, red cell distribution width; Thal, thalassaemia

The retention time alone [n = 4 (*i.e*., HbS, HbD-Punjab, HbQ-India, HbH)] or in conjunction with %Hb [n = 3 (*i.e*., HbJ-Meerut, HbE, δβ-thal trait/HPFH)] or with the peak characteristics [n = 2 (HbD-Iran, Hb-Lepore)] could identify all the 9 haemoglobin variants seen. However, Hb-electrophoresis was resorted to in many doubtful cases to confirm the diagnosis. Family HPLC screening also helped in some difficult cases especially in double heterozygous states. Role of family studies has been emphasized by other studies also^4^.Various normal and variant haemoglobins encountered were as follows:

*Haemoglobin variants with retention times <1.0 min*: One case of HbH disease was detected, which was confirmed by fast-moving band on electrophoresis and a positive HbH inclusion test.

*Haemoglobin variants with retention times in the p1 window (0.63–0.85 min)*: No haemoglobin variants were detected in this window.

*Haemoglobin variants with retention times in the F window (0.98-1.20 min)*: At least seven haemoglobin variants (four β- and three α-variants) are expected to elute in this window, all in quantities >10 per cent^5^. However, most of our patients with high Hb in the F window were mostly homozygous β-thalassaemia patients or double heterozygous β thalassaemia/haemoglobinopathy patients confirmed by Hb-electrophoresis and family studies. Four cases revealed high HbF levels in the range of 1-10 per cent, two of whom had haemolytic anaemia (Coombs test positive) and the other two were pregnant patients. No variant was found in this window.

*Haemoglobin variants with retention times in the p2 window (1.24-1.40 min)*: HbA1c elutes in the P2 window. When the elution peak was >7 per cent of the total haemoglobin, the patient records were checked for indication of diabetes. We came across two cases of known diabetics with a mean P2 value of 13.2 per cent and retention time of 1.28 min. No haemoglobin variants were detected in this window.

*Haemoglobin variants with retention times in the p3 window (1.40-1.90 min)*: A previous study has found nine haemoglobin variants (four α- and five β-variants) with elution peaks in the P3 window^6^. We found a single case of possible HbJ-Meerut which was suspected based on its retention time (1.73 min) and Hb-percentage (26.5%).

*Haemoglobin variants with retention times in the A_0_ window (1.90-3.10 min):* Six haemoglobin variants (two α- and four β-variants) have been reported in the A_0_ window^6^. No such variant was found in our study.

*Haemoglobin variants with retention times in the A_2_ window (3.30-3.90 min)*: Four haemoglobin variants had elution peaks in the A_2_ window- Hb A_2_, HbE, Hb-Lepore and HbD Iran. The retention times and %Hb for HbA_2_ (3.65 min) and HbE (3.73 min) were significantly different (*P*<0.001). The retention time for HbD-Iran (3.62 min) appeared to be different from those of HbE (3.73 min) and Hb Lepore (3.5 min). However, statistical analysis could not be done because of less number of cases. In addition, the mean %Hb of HbD-Iran (40.6%) was greater than either of these Hb (HbE- 27.8%, Hb-Lepore- 16.4%). So the values more than 40 per cent in Hb A_2_ window with a different retention time made us suspect Hb D Iran which was confirmed by starch agarose gel electrophoresis where a band in the SDG region was seen Hb Lepore could be differentiated and identified based on its retention time, %Hb and the characteristic hump on the downward slope of the elution peak^6^. δβ-thalassaemia trait presented with a low HbA_2_ (2.8%) and mildly elevated HbF levels (13.3%).

*Haemoglobin variants with retention times in the D window (3.90-4.30 min)*: Only one haemoglobin variant; HbD-Punjab, with retention times of 4.07 min, was identified in D window. The mean HbA_2_ values for HbD-Punjab trait (1.7 ± 0.4%) were significantly lower (*P*<0.001) than the range for HbA_2_ in the normals.

*Haemoglobin variants with retention times in the S window (4.30-4.70 min)*: Only HbS variant, with retention times of 4.41 min, was seen in this window. The mean HbA_2_ value for HbS trait (3.3 ± 0.3%) was significantly higher (*P*<0.001) than the range for HbA_2_ in the normal samples. A new abnormal haemoglobin Hb D Agri with 2 amino acid substitutions in the same β globin chain [β9 (A6) Ser →Tyr, β121(GH-4) Glu →Gln] has been reported in India which can be mistaken for HbS based on HPLC alone^7^. However, a negative sickling and solubility test should raise a suspicion followed by molecular studies for confirmation.

*Haemoglobin variants with retention times in the unknown window (4.70–4.90 min)*: Three cases of HbQ-India were identified with a retention time of 4.7 ± 0.01 min and mean %Hb of 18.5 ± 0.2%.

*Haemoglobin variants with retention times in the C window (4.90–5.30 min)*: No haemoglobin variant was detected in this window.

*Effect of iron deficiency anaemia on HbA_2_ levels*: The patients with normal HPLC pattern (total 203 in number where iron studies were available) were divided in two groups depending upon their iron profile. The levels of HbA_2_ for 186 patients with IDA (2.74 ± 0.34%) was found to be significantly lower (*P*=0.004) than the 17 patients with normal iron profile (3.03 ± 0.26%). However; there was no significant difference in the levels of HbA_2_ for 25 β-thalassaemia trait patients, 18 with concomitant IDA (5.49 ± 0.52%) and 7 without IDA (5.09 ± 0.58%).

*Effect of megaloblastic anaemia on HbA_2_ levels*: The patients with normal HPLC were divided in two groups depending upon their MCV values (>110fl) and confirmed by further testing. The 19 patients with megaloblastic anaemia showed HbA_2_ levels (3.32 ± 0.56%) which was significantly higher (*P*<0.001) than the HbA_2_ levels (2.89 ± 0.37%) of normal cases with MCV<110fl.

## Discussion

HPLC has been shown to be a sensitive, specific, and reproducible alternative to electrophoresis. With automation and quantitative power, it appears to be a sensitive and accurate technique for direct identification and quantification of normal and abnormal haemoglobin fractions^8^–^13^. Different reports have addressed the precision of the retention times obtained with stored normal^14^ and abnormal samples^8^,^9^. There are a few studies from India which evaluated and emphasized the role of HPLC for diagnosis of thalassaemia and various haemoglobinopathies^3^,^15^.

In this study, apart from β-thalassaemia, nine additional variants were encountered with various incidences; HbS, HbE and HbD-Punjab being the most common variants present. The percentage of variant Hb can generally be the initial predictor whether the detected variant is an α-or β-variant. In our study, all the α-variants had mean %Hb values <27 per cent, whereas among the β-variants, all had mean %Hb values >35 per cent except for HbE (27.8 ± 3.4). The lower levels of HbE compared to other β-chain structural variants could be attributed to HbE being a thalassaemic haemoglobinopathy with unstable m-RNA^16^. In addition, one patient with significantly lower level of HbE (20.5%) was found to have concomitant α- thalassaemia trait confirmed by DNA analysis.

HbBarts, HbH and HbF_1_ (the acetylated form of HbF) were not included in the chromatogram report as HPLC software did not integrate elution peaks that occurred at < 0.63 min. Therefore, these elution peaks were detected only by visual analysis of the plot. The elution peak characteristic in addition to the retention time can be used to identify certain haemoglobin variants, *e.g*., in HbA_2_ window; Hb-Lepore shows a characteristic hump on the downward slope in comparison to HbE, whereas HbD Iran shows a broad based curve which does not touch the baseline as against HbE. Joutovsky *et al*^6^ found statistically significant difference amongst the retention times of different normal and variant haemoglobins within the same retention windows.

Apart from the various variant haemoglobins, the percentages of Hb F and HbA_2_ also have important diagnostic implications. Along with the cases of homozygous β-thalassaemia, elevated HbF levels were encountered in sickle cell disease, various double heterozygous states, δβ-thalassaemia trait/HPFH confirmed by family studies. In addition, some cases (n= 4) showed isolated raised HbF, where no other abnormality related to haemoglobinopathy was found and family study was negative. Such cases were attributed to pregnancy (2 cases) or haemolytic anemia (2 cases). HbA_2_ levels may vary with unrelated haemoglobinopathies as well. HbD-Punjab trait was associated with decreased HbA_2_ (1.4-2.6%) levels, confirming previous reports where a similar range (0.9-2.5%) was reported^17^,^18^. It was postulated that the decrease in HbA_2_ may be attributable to either co-elution with the HbA_0_ or the HbD-Punjab peaks due to integration error or the mutation itself influencing the amount of δ-chain. The mean values of HbA_2_ for HbS trait has been reported to be elevated in previous reports. The reason may be HbS adducts (carbamylated and glycated) coeluting with HbA_2_^19^. In our study, HbA_2_ was found to be significantly higher in HbS trait cases than the range for HbA_2_ in the normals.

HPLC was also able to separately identify two haemoglobin variants of HbD family; HbD-Iran and HbD-Punjab. Both exhibited identical electrophoretic mobilities but eluted in A_2_ and D windows respectively on HPLC. These situations are clinically important because HbD-Punjab produces a significant sickling disorder when present in a double heterozygous HbD- HbS form; whereas HbD-Iran is clinically benign^20^,^21^. The misdiagnosis of HbD-Iran as HbD-Punjab based solely on Hb- electrophoresis or as HbE based solely on Hb- HPLC, where the subtle difference in %Hb or retention time is disregarded, may lead to incorrect genetic counselling in addition to undue anxiety for the family.

The patients with IDA (with a normal HPLC) showed significantly lower levels of HbA_2_ than the patients with normal iron profile as reported by others also^22^. Although previous studies suggest a significant reduction in HbA_2_ levels in thalassaemia trait patients associated with IDA^22^; there was no significant difference in the levels of HbA_2_ for 25 thal trait patients with or without IDA in our study. Similar observation has been made by Madan *et al*^23^. In fact, the level of HbA_2_ was slightly higher in the cases with concomitant IDA than without IDA. It is difficult to render any definite explanation for this discrepant observation especially since routine mutation analysis for these cases were not carried out for the type of mutations. However, the implication that can possibly be derived by this finding is that concomitant iron deficiency in β-thalassaemia trait may not cause significant lowering of HbA_2_ where it may be missed. Patients with megaloblastic anaemia and normal HPLC findings, showed significantly higher HbA_2_ levels than the normals as shown earlier^22^. The importance of nutritional deficiencies on the levels of HbA_2_ is mainly in the patients with borderline raised HbA_2_ levels of 3.5-3.9 per cent. There are several causes of borderline HbA_2_ including β-thalassaemia trait with silent mutations, α-triplication and IDA. A recent study has reported 22.9 per cent positivity for a molecular defect in the β-, δ- or α-globin genes among 410 subjects with borderline HbA_2_ values of 3.1-3.9 per cent^24^.

The present findings show HPLC as an excellent, powerful diagnostic tool for the direct identification of haemoglobin variants with a high degree of precision in the quantification of normal and abnormal haemoglobin fractions. CE-HPLC (β-thal short program) may be a valuable tool in rapid diagnosis of a varied spectrum of haemoglobinopathies. It is diagnostic in most cases and only a few require other modalities for validation. Retention time and percentage of variant haemoglobin can provide important clues in differentiating variant haemoglobins eluting in the same window. Iron deficiency anaemia causes significant lowering of HbA_2_ values and megaloblastic anaemia causes significant elevation of HbA_2_ values in patients with normal HPLC. HbA_2_ in the borderline range needs further evaluation especially for silent mutations, α- thalassaemia and co-existing nutritional deficiency.

## References

[1] (1998). Working Party of the General Hematology Task Force of the British Committee for Standards in Hematology. Guideline: the laboratory diagnosis of hemoglobinopathies. *Br J Hematol*.

[2] Bain BJ (2001). *Hemoglobinopathy diagnosis*.

[3] Tyagi S, Saxena R, Choudhry VP (2003). HPLC - how necessary is it for haemoglobinopathy diagnosis in India?. *Indian J Pathol Microbiol*.

[4] Rangan A, Handoo A, Sinha S, Saxena R, Verma IC, Kumar S (2009). Utility of family studies in diagnosing abnormal hemoglobins/thalassemic states. *Indian J Pediatr*.

[5] Hardison RC, Chui DHK, Giardine B, Riemer C, Patrinos GP, Anagnou N (2002). HbVar: a relational database of human hemoglobin variants and thalassemia mutations at the globin gene server. *Hum Mut*.

[6] Joutovsky A, Hadzi-Nesic J, Nardi MA (2004). HPLC retention time as a diagnostic tool for hemoglobin variants and hemoglobinopathies: A study of 60000 samples in a clinical diagnostic laboratory. *Clin Chem*.

[7] Colah RB, Wadia M, Surve R, Nadkarni A, Phanasgaonkar S, Gorakshakar A (2001). Hb D Agri [b9 (A6) Ser ® Tyr, b 121 (GH – 4) Glu ® Gln]: A new Indian hemoglobin variant with two amino acid substitutions in the same beta chain. *Hemoglobin*.

[8] Ou CN, Rognerud CL (2001). Diagnosis of hemoglobinopathies: electrophoresis vs. HPLC. *Clin Chim Acta*.

[9] Riou J, Godart C, Didier H, Mathis M, Bimet C, Bardakdjian-Michau J (1997). Cation-exchange HPLC evaluated for presumptive identification of hemoglobin variants. *Clin Chem*.

[10] Eastman JW, Wong R, Liao CL, Morales DR (1996). Automated HPLC screening of newborns for sickle cell anemia and other hemoglobinopathies. *Clin Chem*.

[11] Eastman JW, Lorey F, Arnopp J, Currier RJ, Sherwin J, Cunningham G (1999). Distribution of hemoglobin F, A, S, C, E and D quantities in 4 million newborn screening specimens. *Clin Chem*.

[12] Mario N, Baudin B, Aussel C, Giboudeau J (1997). Capillary isoelectric focusing and high-performance cation-exchange chromatography compared for qualitative and quantitative analysis of hemoglobin variants. *Clin Chem*.

[13] Fucharoen S, Winichagoon P, Wisedpanichkij R, Sae-Ngow B, Sriphanich R, Oncoung W (1998). Prenatal and postnatal diagnoses of thalassemias and hemoglobinopathies by HPLC. *Clin Chem*.

[14] Mario N, Baudin B, Aussel C, Giboudeau J (1997). Capillary isoelectric focusing and high-performance cation-exchange chromatography compared for qualitative and quantitative analysis of hemoglobin variants. *Clin Chem*.

[15] Colah RB, Surve R, Sawant P, D’Souza E, Italia K, Phanasgaonkar S (2007). HPLC studies in hemoglobinopathies. *Indian J Pediatr*.

[16] Pignatti CB, Galanello R, Greer JP, Foerster J, Lukens JN, Rodgers GM, Paraskevas F, Glader B (2004). Thalassemias and related disorders: quantitative disorders of hemoglobin synthesis. *Wintrobes clinical hematology*.

[17] Cotton F, Gulbis B, Hansen V, Vertongen F (1999). Interference of hemoglobin D in hemoglobin A_2_ measurement by cation-exchange HPLC. *Clin Chem*.

[18] Dash S (1998). HbA_2_ in subjects with HbD. *Clin Chem*.

[19] Suh DD, Kraus JS, Bures K (1996). Influence of hemoglobin S adducts on hemoglobin A_2_ quantification by HPLC. *Clin Chem*.

[20] Nagel RL, Steinberg MH, Steinberg MH, Forget BG, Higgs DR, Nagel RL (2001). Hemoglobin SC disease and HbC disorders. *Disorders of hemoglobin: genetics, pathophysiology, and clinical management*.

[21] Steinberg MH, Steinberg MH, Forget BG, Higgs DR, Nagel RL (2001). Compound heterozygous and other sickle hemoglobinopathies. *Disorders of hemoglobin: genetics, pathophysiology, and clinical management*.

[22] Alperin JB, Dow PA, Petteway MB (1977). Hemoglobin A_2_ levels in health and various hematologic disorders. *Am J Clin Pathol*.

[23] Madan N, Sikka M, Sharma S, Rusia U (1998). Phenotypic expression of hemoglobin A2 in beta-thalassemia trait with iron deficiency. *Ann Hematol*.

[24] Giambona A, Passarello C, Vinciguerra M, Li Muli R, Teresi P, Anzà M (2008). Significance of borderline hemoglobin A2 values in an Italian population with a high prevalence of b-thalassemia. *Haematologica*.

